# A neural signature for combined action observation and motor imagery? An fNIRS study into prefrontal activation, automatic imitation, and self–other perceptions

**DOI:** 10.1002/brb3.2407

**Published:** 2022-01-07

**Authors:** Jonathan R. Emerson, Matthew W. Scott, Paul van Schaik, Natalie Butcher, Ryan P. W. Kenny, Daniel L. Eaves

**Affiliations:** ^1^ School of Health and Life Sciences Teesside University Middlesbrough UK; ^2^ Department of Psychology Faculty of Health, Psychology and Social Care Manchester Metropolitan University Manchester UK; ^3^ Department of Psychology School of Social Sciences, Humanities & Law Teesside University Middlesbrough UK; ^4^ Population Health Sciences Institute Faculty of Medical Sciences Newcastle University Newcastle UK

**Keywords:** action planning and control, demonstration, mental practice, perceived closeness, self–other distinction, social cognition

## Abstract

**Introduction:**

Research indicates that both observed and imagined actions can be represented in the brain as two parallel sensorimotor representations. One proposal is that higher order cognitive processes would align these two hypothetical action simulations.

**Methods:**

We investigated this hypothesis using an automatic imitation paradigm, with functional near‐infrared spectroscopy recordings over the prefrontal cortex during different motor simulation states. On each trial, participants (n = 14) observed a picture of a rhythmical action (instructed action) followed by a distractor movie showing the same or different action. Participants then executed the instructed action. Distractor actions were manipulated to be fast or slow, and instructions were manipulated during distractor presentation: action observation (AO), combined action observation and motor imagery (AO+MI) and observe to imitate (intentional imitation). A pure motor imagery (MI) condition was also included.

**Results:**

Kinematic analyses showed that although distractor speed effects were significant under all instructions (shorter mean cycle times in execution for fast compared to slow trials), this imitation bias was significantly stronger for combined AO+MI than both AO and MI, and stronger for intentional imitation than the other three automatic imitation conditions. In the left prefrontal cortex, cerebral oxygenation was significantly greater for combined AO+MI than all other instructions. Participants reported that their representation of the self overlapped with the observed model significantly more during AO+MI than AO.

**Conclusion:**

Left prefrontal activation may therefore be a neural signature of AO+MI, supporting attentional switching between concurrent representations of self (MI, top‐down) and other (AO, bottom‐up) to increase imitation and perceived closeness.

## 1 INTRODUCTION

Action observation (AO) refers to the deliberate and structured observation of human actions (Neuman & Gray, 2013), whereas motor imagery (MI) is defined as “the mental representation of action without any [overt] concomitant body movement” (Guillot & Collet, 2008, p. 31). Although the brain areas involved in AO at least partially overlap with those involved in MI, these brain regions (namely, the bilateral ventral premotor, dorsal premotor, pre‐SMA, and parietal regions) are also recruited during motor execution (Hardwick et al., 2018). Jeannerod's (2006) influential hypothesis suggested both AO and MI can be regarded as two forms of motor simulation that are functionally equivalent to each other. Despite this early integrative account, these two forms of motor simulation have traditionally been studied either in isolation from each other or compared in terms of their impact on motor skills (e.g., Gatti et al., 2013; see Vogt et al., 2013). More recently, a growing body of research demonstrates the advantages of instructing combined action observation and motor imagery (AO+MI) instructions on motor learning (Marshall, Wright, Holmes, & Wood, 2020; Romano‐Smith et al., 2019, 2018) and neurophysiological activity when undertaking AO+MI (Macuga & Frey, 2012; Villiger et al., 2013). Combined AO+MI typically involves participants imagining the kinesthetic experience and sensations of an action, while at the same time also observing a visual display of the same action (Eaves, Riach, et al., 2016). This form of mental practice therefore requires participants to synchronize the sensorimotor representation of their imagined action with a representation of the observed movement in real time (Emerson et al., 2018). One proposal is that higher order (top‐down) cognitive processes are necessary for aligning these two hypothetical action simulations (Eaves, Behmer, et al., 2016; Eaves, Riach, et al., 2016). In the present study, we employed an automatic imitation paradigm to investigate this hypothesis, with functional near‐infrared spectroscopy (fNIRS) recordings obtained over the prefrontal cortex during different motor simulation states.

Behavioral research across a range of tasks and paradigms has demonstrated significant benefits in motor development for combined AO+MI instructions when compared to either AO or MI instructions (e.g., Marshall, Wright, Holmes, & Wood, 2020; Scott et al., 2018; Taube et al., 2014). Throughout a series of studies, combined AO+MI instructions have also been assessed in terms of their impact on automatic imitation effects in healthy adults (Eaves et al., 2014, 2012) and children (Scott et al., 2019). Automatic imitation is a type of stimulus–response compatibility effect whereby observing a task‐irrelevant action can facilitate execution of similar and impede execution of different actions (Cracco et al., 2018; Heyes, 2011). For example, movement initiation times are shorter when executing hand opening while also observing a hand opening compared to when observing a hand closing. Although most studies have used reaction time measures to quantify automatic imitation effects (Ramsey, 2018), Eaves et al. (2012) were the first to establish that observing a rhythmical distractor action at either a fast or slow pace across trials significantly biased execution speeds in subsequent rhythmical action execution. This *imitation bias* was also present when the action type and plane were incompatible between the instructed and observed actions (e.g., “plan to execute vertical face washing while you observe horizontal painting”). The imitation bias for incompatible trials represents a genuine automatic imitation effect, because this was when the distractor action was not functionally relevant to motor planning.

Using the same paradigm in two subsequent studies, a robust imitation bias was obtained for both AO and MI separately, whereas combined AO+MI instructions significantly increased this bias (Eaves et al., 2014; Eaves, Behmer, et al., 2016). Using electroencephalography (EEG) recordings, Eaves, Behmer, et al. (2016) confirmed the AO+MI instruction also significantly increased event‐related desynchronization (ERD) of the mu rhythm over the primary motor cortex. The same pattern of neurophysiological results has also been reported in studies using a range of tasks and measures, including functional magnetic resonance imaging (fMRI; e.g., Macuga & Frey, 2012; Nedelko et al., 2012; Taube et al., 2015; Villiger et al., 2013), transcranial magnetic stimulation (TMS; e.g., Bruton et al., 2020; Mouthon et al., 2015; Sakamoto et al., 2009; Tsukazaki et al., 2012; Wright et al., 2016, 2014, 2018), and fNIRS (Holper et al., 2012, 2010).

So far, research into the neurophysiological effects of combined AO+MI has focused primarily on the neural mechanisms involved in motor planning and motor output (see Emerson et al., 2018). Research is yet to examine the impact of this instruction on the neural substrates that support a broader range of sensorimotor‐related processes, such as social cognition. In a natural social setting, the ability to distinguish between the self and other people is essential for inhibiting the automatic tendency to imitate observed actions (Brass et al., 2009). This is because the observer must continuously distinguish between their own motor plans and the actions of observed agents (see Brass & Heyes, 2005). Although this self–other distinction was a core component of the task for participants in the aforementioned automatic imitation paradigm, it is currently unclear if AO+MI instructions additionally modulate self–other perceptions.

To our knowledge, Eaves, Behmer, et al. (2016) is the only study to date that has explored the neural correlates of AO+MI in brain areas related to higher order cognitive process. Their EEG study revealed that compared to both AO and MI, the combined AO+MI instruction produced significantly stronger ERD over the left rostral prefrontal cortex (BA 10). A primary role for the rostral prefrontal cortex is to route attention between information arising from sources either within the body (i.e., stimulus‐independent) or within the environment (i.e., stimulus‐orientated), without being directly involved in any domain‐specific processing per se (Burgess et al., 2007, 2006; Eaves, Riach, et al., 2016). As suggested by Eaves, Riach, et al. (2016), this “gateway hypothesis” for attentional processes would therefore predict increased neural activity in rostral prefrontal areas specifically for synchronized AO+MI. This is because the AO+MI instruction requires ongoing reallocations of attention, or “switching,” between the externally induced AO simulation and the internally generated MI components. On the basis of their EEG data, Eaves, Riach, et al. (2016) and Eaves, Behmer, et al. (2016) proposed that both an observed and imagined action might theoretically be represented as two parallel sensorimotor streams. Those authors suggested that the prefrontal cortex would be involved in aligning the action simulations that are involved in representing both the self and the observed other person. Here, we sought further evidence of this proposal using fNIRS, as an alternative and low‐cost neuroimaging technique.

fNIRS is a method for quantifying cerebral oxygenation changes in oxy‐hemoglobin (HbO) and deoxy‐hemoglobin (HHb) concentrations. These changes correlate strongly with the blood‐oxygen‐level‐dependent (BOLD) signal recorded in fMRI (Steinbrink et al., 2006). Previous fNIRS studies have assessed cerebral oxygenation levels for AO (Shimada & Oki, 2012), MI (Kober & Wood, 2014), and for action execution compared to AO (Balconi & Cortesi, 2016), MI (Ishizu et al., 2009), or AO and MI (Balconi et al., 2017). Those studies largely replicate previous fMRI findings (e.g., Filimon et al., 2015; Rizzolatti & Sinigaglia, 2010), showing greater cortical activation across sensorimotor and parietal regions for AO and MI than for baselines, with greater activations found overall for execution (e.g., Holper et al., 2010; Króliczak, 2013).

In the present study, we adopted the automatic imitation paradigm employed by Eaves and colleagues (Eaves et al., 2014, 2012; Eaves, Behmer, et al., 2016). A unique feature of our experiment was that, for the first time, fNIRS was used to assess cerebral oxygenation in the prefrontal cortex for four separate instruction conditions: combined AO+MI compared to AO, MI, and intentional imitation (also referred to as “observe to imitate”). A within‐participants design was used to compare these four instruction conditions in a single experiment, which has also not been done previously. The initial analysis of the behavioral data represents an important prerequisite for subsequent interpretation of the fNIRS analyses. In line with previous research, which involved comparisons across groups of participants in three different experiments (Eaves et al., 2014, 2012; Eaves, Behmer, et al., 2016), our prediction was that the imitation bias would be stronger for the combined AO+MI instruction than for both the AO and MI instructions, and that this bias would be significantly greater for intentional imitation than for the three automatic imitation conditions (i.e., combined AO+MI, AO, and MI).

In accordance with Eaves, Behmer, et al.’s (2016) EEG study of electrophysiological activity in the prefrontal cortex, we predicted AO+MI would produce significantly greater cerebral oxygenation in the left prefrontal cortex than AO and MI, due to the increased requirement for attentional switching during AO+MI. In the present study, the AO condition involved motor preparation and execution, whereas the AO condition in Eaves, Behmer, et al. (2016) study did not involve execution. We further predicted that cerebral oxygenation would be significantly higher for AO+MI than for intentional imitation. Although this has not previously been tested, attentional switching may be reduced in intentional imitation because of an increased weighting of attention toward external sources of information for motor planning.

Previous research has shown that the neural correlates involved in monitoring our own actions are comparable to those involved in monitoring the actions of others (see Miltner et al., 2004; Shane et al., 2008; van Schie et al., 2004). Therefore, alongside the behavioral and neural correlates, we assessed self–other perception across the AO, AO+MI, and intentional imitation conditions. Greater perceived overlap between the self and other has been associated with increased empathic responding (Cialdini et al., 1997), perspective‐taking (Aron et al., 1991), and fostering social bonds (Galinsky et al., 2005). Although this measure may not have been previously used within automatic imitation paradigms, behavioral mimicry research has shown a positive relationship exists between imitation and affiliation (Chartrand & van Baaren, 2009). We therefore predicted a greater sense of self–other overlap would be found for the intentional imitation condition than for the other two conditions, with a greater overlap for AO+MI than for the AO condition.

## 2 METHOD

### 2.1 Participants

Participants (*n *= 14, mean age = 23 years [SD = 6.7], age range = 18–37 years, *n* = 6 males) were recruited all having normal or corrected to normal vision. Participants included were naïve to the study's purpose, all right‐hand dominant (Edinburgh Handedness Inventory; Oldfield, 1971), and without physical injuries or any known history of mental health conditions. Written informed consent was acquired prior to participation, and ethical approval had been granted by the local ethics committee.

A priori power analyses were conducted using G*Power (Faul et al., 2007). The effect size used for this analysis was based on Eaves et al.’s (2014) study that used the same paradigm and stimuli as in the current study and demonstrated a strong effect ($\eta_{p}^{2}$ = 0.6) for the same instruction condition. A repeated‐measures ANOVA was used as the basis for the assumptions with a significance level of 0.05 and power of (1 − β) = 0.80. Following this analysis, the total number of participants required to observe an effect size of *f* = 1.20 (equivalent to $\eta_{p}^{2}=0.6$) was *n* = 4. The sample used in the present study (*n* = 14) was therefore considered sufficient to observe such an effect in the kinematic data. This sample size is also comparable to other studies that used between eight and 15 participants when investigating fNIRS measures in response to AO, MI, and imitation conditions (Holper et al., 2010, 2013).

### 2.2 Task and design

**FIGURE 1:**
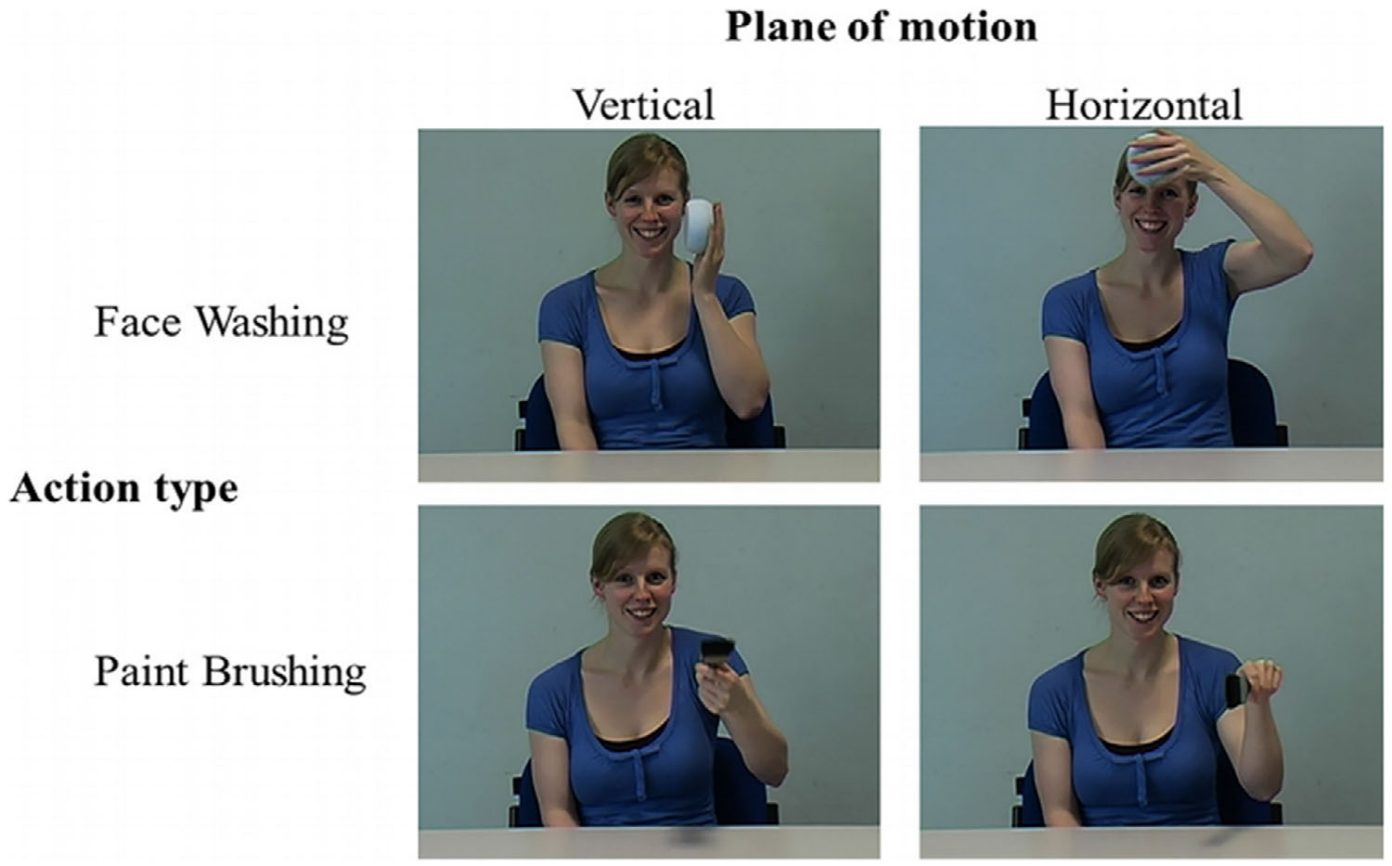
Instructed action stimuli with the factors of action type and plane of motion

**FIGURE 2:**
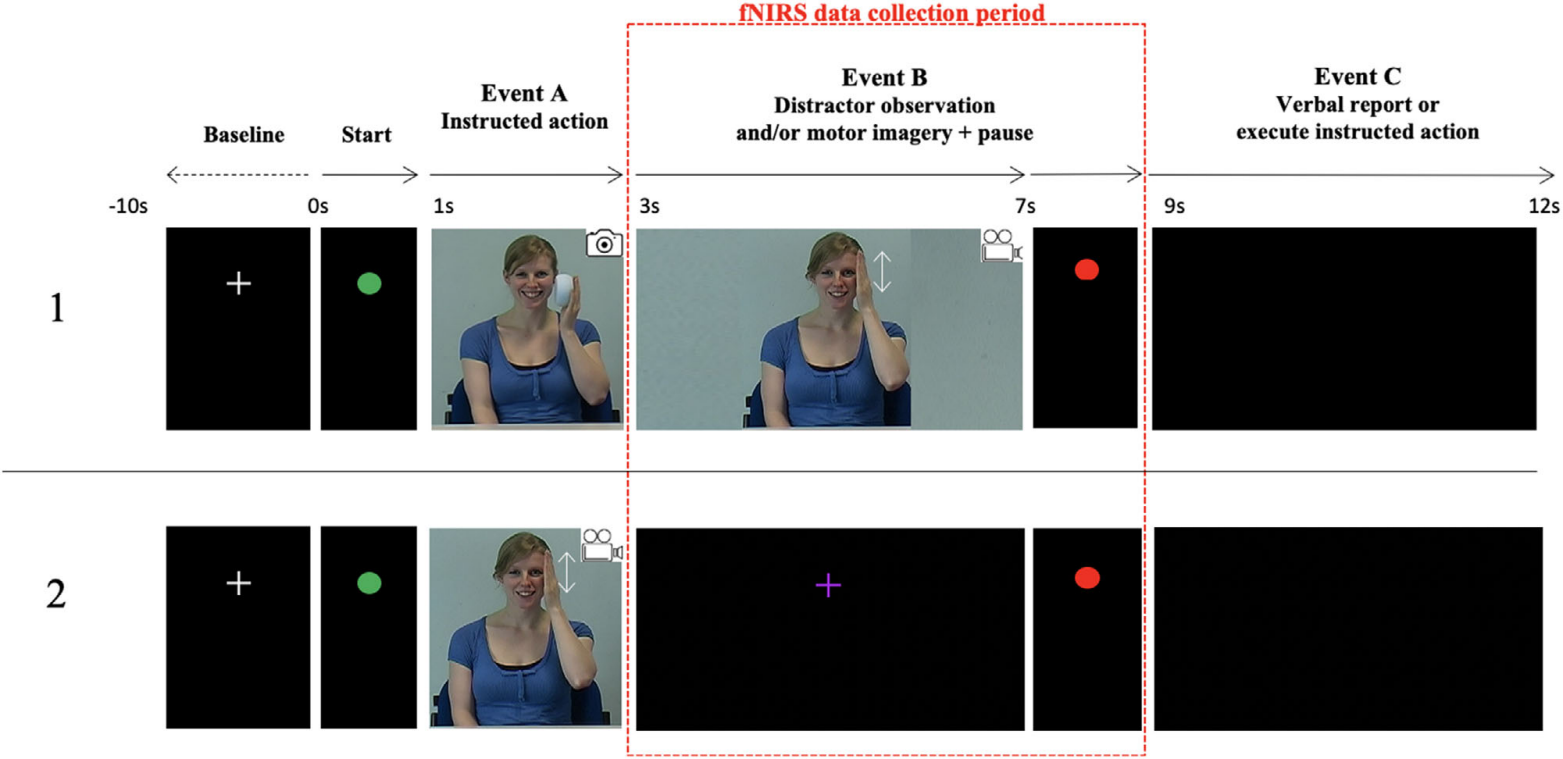
Participants began each trial by pressing the keyboard space bar. Next, they observed a green “get ready” cue for 1 s, followed by a picture (Event A) of the instructed action (either face washing or paint brushing in either the vertical or horizontal plane). This was followed by observation and/or imagery for 4 s (Event B) before participants executed the instructed action for 4 s (Event C) either at their own preferred speed or as close as possible to the distractor speed (intentional imitation condition only). The main fNIRS capture period was recorded over the prefrontal cortex throughout Event B. Movement kinematics were recorded throughout Event C. The same trial structure (row 1) was used for the following three instruction conditions: action observation (AO), combined action observation and motor imagery (AO+MI), and intentional imitation. A modified trial structure was used for the MI condition (row 2). For AO, participants were instructed to “watch the girl's face” during Event B. For combined AO+MI, participants were told to “imagine performing the instructed action in time with the observed distractor action.” For intentional imitation, participants were asked to “copy the distractor speed as closely as possible” during Event C. In these three conditions, participants verbally reported the match between the instructed and distractor actions on 25% of the trials. For the block of MI trials (row 2), participants observed a 2‐s movie of the instructed action (Event A), after which they were asked to “imagine performing the instructed action while fixating on the purple cross” (Event B). They then executed the instructed action at their own preferred speed during Event C

In each experimental trial, participants observed a picture of an everyday rhythmical action (instructed action), followed by a short movie (distractor action) of either the same or a different action (see Figures 1 and 2). They then executed the instructed pantomime action. Across trials, slow and fast versions of each distractor action were used. This experimental paradigm is well established in previous studies (Eaves et al., 2014, 2012; Scott et al., 2019).

The four dependent measures included the mean response cycle time (ms), the ratio between slow and fast distractor trials (%), a compound cerebral oxygenation score (oxy), and self–other perceptions. The factor of instruction condition was manipulated within each dependent variable, with four levels (AO, MI, AO+MI, and intentional imitation). As in previous research (e.g., Scott et al., 2019), this factor was manipulated across four blocks of 16 trials. Although this was the only factor assessed in the self–other perception data, in the cycle time data the distractor speed (slow, fast; ms data only) and the compatibility between the instructed and distractor actions (same, different action, % data only) were manipulated within each block. In the oxy data, the factor of hemisphere (left, right) was manipulated within blocks, wherein the data were pooled across the factors of distractor speed and compatibility for this measure.

### 2.3 Stimuli and apparatus

The instructed picture‐and‐distractor movie stimuli were created using a digital video camera (Panasonic NV‐MX500B). The two instructions within these stimuli were face‐washing and paint‐brushing performed in both the vertical and horizontal planes (see Figure 1).

Data were pooled across the four instructed actions. The primary interest was in the compatibility between distractor action and instructed action as opposed to separate effects of the independent variables for each individual action. Spatial compatibility is known to facilitate imitation in comparison to anatomically coordinated but spatially conflicting information (Buccino et al., 2004). Therefore, all actions performed by the model were done so using their left hand to provide a mirrored image of the actions that were then performed by participants using their right hand (e.g., Buccino et al., 2004; Scott et al., 2019). Eight distractor movies were used demonstrating the two instructed actions with varied speed (one slow and one fast) and plane (one horizontal and one vertical). Although the model's performance was aligned using an auditory metronome to speeds of 60 and 90 beats per minute (bpm) during filming, the videos used in the main experiment were presented without sound. Instructed picture actions were displayed including the relevant object (sponge or paintbrush), facilitating quick discrimination between actions. Participants performed pantomimed actions without objects and therefore were not required to select the associated object at the beginning of each trial. Pantomimed actions were also used in the distractor movies to enable participants to easily distinguish between distractor and instructed stimuli and to have greater similarity with the executed action.

Stimuli were displayed on a 17‐inch LCD (Hewlett Packard) computer screen and presented against a black background using Superlab 4.5 software (Cedrus Corp.). Participants were seated approximately 80 cm away from the screen, at a desk in a dimly‐lit room. At the start of each trial, the participants’ right hand was positioned on a black cross located on the desk 20 cm ahead of them. The participants’ kinematic data were recorded using a magnetic motion sensor fitted to the distal end of the second metacarpal bone of the right hand, sampled at 103 Hz in three‐dimensional space for 4‐s periods (Minibird Magnetic Tracking System, Ascension Technologies), and data were stored on a separate PC.

Brain imaging signals were captured via a fNIRS imaging system (FNIR400, Biopac Systems Inc.), which recorded the changes in HbO and HHb as well as total hemoglobin (Total‐Hb) relative to baseline recordings. The basis for using fNIRS to measure cortical activity is derived from the interaction between neuronal (electrical) activity and associated hemodynamic changes known as neurovascular coupling (Vanzetta & Grinvald, 2008). The fNIRS sensor consisted of four light emitting diodes (LEDs) and 10 photodetectors situated on a flexible circuit board. The LEDs (in conjunction with each surrounding photodetector) generated a total of 16 optodes (i.e., channels of data) that were positioned in two parallel rows of eight optodes across the forehead.

**FIGURE 3:**
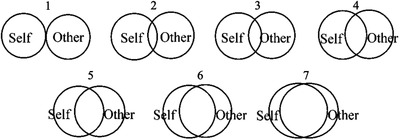
Inclusion of the other in the self (IOS) scale. Participants verbally reported the number relating to the pair of circles that most accurately represented their general perception of the interconnectedness between them self and the observed model (1 being the least up to 7 being the most interconnected)

Perceived closeness between the self and other was also assessed using the self‐report scale “inclusion of the other in self” (IOS; Aron et al., 1992) after three of the four blocks of trials (AO, AO+MI, and intentional imitation). This is a useful nonspecific measure for assessing the general sense of being interconnected (i.e., closeness) with another person. This is expressed in terms of the overlapping representations of the self and interacting partners. The IOS scale allowed participants to report the degree to which they perceived their self was independent from or interconnected with the observed other (see Figure 3). This measure was excluded from the MI block due to the lack of visual stimulus for comparison.

To screen for motor imagery ability, participants completed the Movement Imagery Questionnaire‐3 (MIQ‐3; Williams et al., 2012). This involved executing overt actions followed by imagined actions. Participants then self‐reported the ease with which they could generate these imagined actions (1 = *very hard*; 7 = *very easy*) on three subscales: visual internal, visual external, and kinesthetic imagery (see Table 1).

**TABLE 1:** Mean scores for the Motor Imagery Questionnaire‐3 with standard deviations in three imagery subcategories

Movement Imagery Questionnaire‐3 subcategory		
Internal visual imagery	External visual imagery	Kinesthetic imagery
5.9 ± 0.4	5.8 ± 0.4	5.7 ± 0.3

### 2.4 Procedure

Participants were familiarized with all parts of the task prior to their involvement in the main experiment (see Scott et al., 2019). Distractor speed was the core manipulation within the current study, with a ratio between slow (60 bpm) and fast (90 bpm) trials of 150%. Participants were not informed of the changes in distractor speed. Participants’ attention to the distractor movie was ensured by asking them to verbally report the match between the instructed and distractor actions (same or different), as well as the properties of the distractor movie (action type and plane of motion) four times per block in a pseudorandom order. As in previous research (e.g., Eaves et al., 2014; Eaves, Behmer, et al., 2016), a single warm‐up trial was included at the start of each block, which was identical to the other trials in the block, but removed from the analysis. A 5‐min rest period was provided between blocks.

#### 2.4.1 Action observation

Trials started once participants pressed the space bar key and a green circle was then displayed for 1 s as a cue to “get ready” (see Figure 2). Following this, a picture of the “instructed” to‐be‐pantomimed action was displayed for 1.5 s (Event A) and then a distractor movie of the same actor pantomiming either the same or a different rhythmical action was shown for 4 s (Event B). This structure replicated the approach taken in previous studies (e.g., Eaves et al., 2014; Scott et al., 2019). Throughout the movie, participants were required to fixate on the model's face to minimize any possible visual coupling to the model's rhythmical arm actions (Schmidt et al., 2007). Within the AO condition, participants were told the distractor action was irrelevant to their task. Rather, they were told the task was a memory game and that they should prepare to execute the instructed action regardless of the distractor action shown in the movie. Following distractor movie offset and after a short pause to allow for fNIRS data collection, participants executed the instructed action at their own preferred speed while movement kinematics were recorded in three dimensions (Event C). The end of the 4‐s recording period was denoted by a computer‐generated auditory signal, after which participants could verbally report distractor properties before returning their hand to the starting location.

The AO block was presented first because it was important to try to reduce the likelihood of participants engaging in spontaneous or deliberate MI during this condition (cf. Wright et al., 2018). Providing imagery training before this block might have encouraged this possibility, by virtue of the imagery instructions themselves. Withholding this training prior to the block instead aimed to promote a more naturalistic and perhaps passive form of AO (cf. Eaves et al., 2012; Scott et al., 2019). The presentation order for the subsequent MI and AO+MI blocks was then fully counterbalanced across all participants.

Before undertaking the three subsequent blocks of trials, participants completed the MIQ‐3 (Williams et al., 2012). An imagery script based on the Physical, Environment, Task, Timing, Learning, Emotion, and Perspective (PETTLEP) principles (Holmes & Collins, 2001) was then read aloud. The main instruction was to mentally simulate the “physical” effort and sensation involved in performing the movement “task” from a first‐person “perspective,” adopting a similar physical seated positioning but without performing any actual movement. Participants were instructed to simulate their performance in real “time” and within their current “environment,” while including any “emotions” typically associated with this performance. These scripts were designed to help participants generate a vivid imagery experience involving all aspects of the task. They were also designed to foster “learning” by increasing the complexity and clarity of the imagery during the intervention period. Participants were then trained to perform MI during AO or MI in the absence of AO, depending on the counterbalanced presentation order for these two blocks.

#### 2.4.2 Combined AO and MI

Participants observed a picture of the instructed action for 1.5 s (Event A), followed by the distractor action movie for 4 s showing either the same or a different action (Event B). During observation of the distractor, participants imagined from a first‐person perspective both the physical sensations and effort involved in performing a dynamic version of the instructed action with their right hand in synchrony with the display (cf. Eaves et al., 2014; Eaves, Riach, et al., 2016; Scott et al., 2019). Each trial ended with participants executing the instructed action for 4 s at their own preferred pace (Event C) before periodically reporting the distractor properties (four times each block) before then starting the next trial.

#### 2.4.3 Motor imagery

Participants first observed a movie of the instructed action for 2 s (Event A), before observing a purple fixation cross for 4 s (Event B). During Event B, participants imagined from a first‐person internal perspective, with their eyes open, the physical sensation and effort involved in executing the instructed action with their right hand at the pace of the preceding movie. Finally, the appearance of a black screen for 4 s (Event C) cued participants to perform the instructed action at their own preferred pace, and during this movement kinematic data were recorded. As in the other conditions, an auditory tone signaled the end of the trial, and participants could return to the starting position for the next trial. Within this condition only, it was not possible to manipulate the compatibility between the instructed action and distractor actions. The duration (1.5 s) of the picture in the other conditions served to increase the verbal task difficulty in detecting differences between the actions for the instructed and distractor action. For the MI condition, the video duration lasted 2 s to ensure that complete movement cycles were displayed regardless of distractor speed.

#### 2.4.4 Intentional imitation

The trial structure for intentional imitation was identical to AO and AO+MI conditions. Action execution (Event C), however, required participants to imitate the cycle time shown in the distractor movie as closely as possible. As such, this instruction was administered last ensuring participants remained naïve to the manipulations of distractor speed in the preceding unintentional imitation instructions.

### 2.5 fNIRS data

Brain imaging signals were captured via a fNIRS imaging system (FNIR400, Biopac Systems Inc.), which recorded the changes in HbO and HHb as well as Total‐Hb relative to baseline recordings. Prior to each trial, baseline activation levels were recorded for 10 s, which has previously been recommended (Albinet et al., 2014; Makizako et al., 2013; Wang et al., 2013). A baseline capture period between 0 and 30 s is recognized as appropriate for reducing the signal‐to‐noise ratio (Pellicer & del Carmen, 2011). During each trial, we also adopted an fNIRS data capture period of 7 s (Event B; see Figure 2). This began at stimulus onset (i.e., distractor video presentation) for 4 s and continued during a 3‐s pause, which was cued on screen by the onset of a red dot. fNIRS data capture ended when the red dot disappeared, which also cued movement execution (Event C). This capture period aligns with previous research that used capture periods of 4–11 s (Byun et al., 2014; Matsuda et al., 2017; Yanagisawa et al., 2010), 6–9 s (Hyodo et al., 2016), and 6–10 s after stimulus onset (Ochi et al., 2018), whereas 6–8 seconds after trial onset has been used for HbO and 7–9 s after trial onset for HHb (Hyodo et al., 2012). Previous behavioral research using this automatic imitation paradigm has also shown the automatic imitation effect persists in movement kinematics over an 8‐s period between distractor movie offset and movement execution onset (Eaves et al., 2012).

The fNIRS data were analyzed offline through the platform fNIRSoft, developed by Ayaz et al. (2010). All data were first filtered using a finite impulse response linear phase low‐pass filter to reduce high‐frequency noise, respiration, and cardiac effects (Ayaz et al., 2010; Izzetoglu et al., 2007). All data were subjected to a sliding‐window motion artifact rejection (SMAR) algorithm to remove motion artifacts and saturated channels (Ayaz et al., 2010). Once the data were filtered, HbO and HHb were calculated using the modified Beer–Lambert law. The data were collapsed across two factors that were manipulated within each instruction condition (i.e., distractor speed and distractor compatibility), providing *n* = 16 trials per instruction condition. Through the use of digital markers, segments during the motor simulation phase of each trial were extracted using synchronization markers before the segments were averaged according to condition (AO, AO+MI, MI, and Intentional Imitation; see Figure 2).

Research shows HHb is sensitive to local hemodynamic changes, less prone to influence from psychophysiological noise, such as heart rate or breathing, and has a close association with the BOLD signal in fMRI (Kreplin & Fairclough, 2013). HbO is less sensitive to probe placement variability, in response to head shape and size. HbO activation is also a more global measure of activation than HHb (Hoshi, 2005; Plichta et al., 2006; Wobst et al., 2001). We therefore calculated a compound score for oxygenation (oxy = HbO – HHb) to capture both measures while controlling for changes in blood volume (cf. Ayaz et al., 2010; Kreplin & Fairclough, 2013). Given the presence of AO in each instruction condition, the “pure” AO condition represents a meaningful level of prefrontal activation (oxy) within the experimental paradigm. Oxygenation for the other three instruction conditions was therefore expressed as a change score relative to the AO condition.

### 2.6 Movement kinematic data

Similar to previous research (see Eaves et al., 2014, 2012; Scott et al., 2019), a custom‐made signal processing application created in Microsoft Visual Studio was first used to calculate mean cycle times (ms) between peak movement kinematic positions. This applied a 6‐Hz low‐pass, second‐order, bidirectional Butterworth filter to smooth the data. For all actions, the data point first taken was the peak maximum of the second movement cycle. The first cycle was not included because this may have also reflected the spatial positioning of the hand before reaching a stable workspace. Mean cycle time was then calculated across peak positions obtained within the 2‐s time window across all conditions. This typically resulted in two to four complete cycles. All trials including invalid responses (incorrect or no action) were discarded (*n* = 13, i.e., 1.5% of all trials undertaken). Two dependent measures were derived from the kinematic data: the mean response cycle time (ms) and ratio (%) between slow and fast distractor trials.

For economy of exposition, we limited the analysis of the movement kinematic data (ms) to one factor of interest, specifically the distractor speed effect (cf. Eaves et al., 2014; Eaves, Behmer, et al., 2016; Scott et al., 2019). All other factors were then analyzed using the cycle time ratio data. Analyses were conducted using R (R Core Team, 2021). These were also formally subjected to assumption testing for any violation of sphericity using Mauchly's test (Mauchly, 1940). The significance level was set to 0.05, and effect sizes were calculated as generalized eta squared values ($n{\overset{ˇ}{G}}^{2}$) or Cohen's *d* (Bakeman, 2005; Cohen, 1992; Olejnik & Algina, 2003). Significant main effects were further investigated using pairwise comparisons with Bonferroni corrections applied.

## 3 RESULTS

### 3.1 Movement kinematics

The overall mean response cycle times (ms) were subjected to a paired samples *t*‐test comparing the two distractor speeds (fast vs. slow). The ratio data (%) were first collapsed across the compatibility factor to run a one‐way ANOVA assessing the factor of instruction (AO vs. MI vs. AO+MI vs. intentional imitation), and then a two‐factorial ANOVA examining the effects of instruction and compatibility with the MI condition removed (because this condition did not include the compatibility manipulation).

#### 3.1.1 Cycle time data (ms)

Analyzing the mean response cycle times (ms) over all experimental conditions revealed a significant difference between the two distractor speeds (*t*(14) = −8.2, *p *< .001, *d *= 3.9), wherein mean cycle times were shorter for fast compared to slow trials (813 vs. 980 ms, respectively). Paired samples *t*‐tests confirmed this distractor speed effect within each compatibility condition for AO, AO+MI, and intentional imitation (all *p*s ≤ .05) and for the single MI instruction condition.

#### 3.1.2 Cycle time data (%)

**FIGURE 4:**
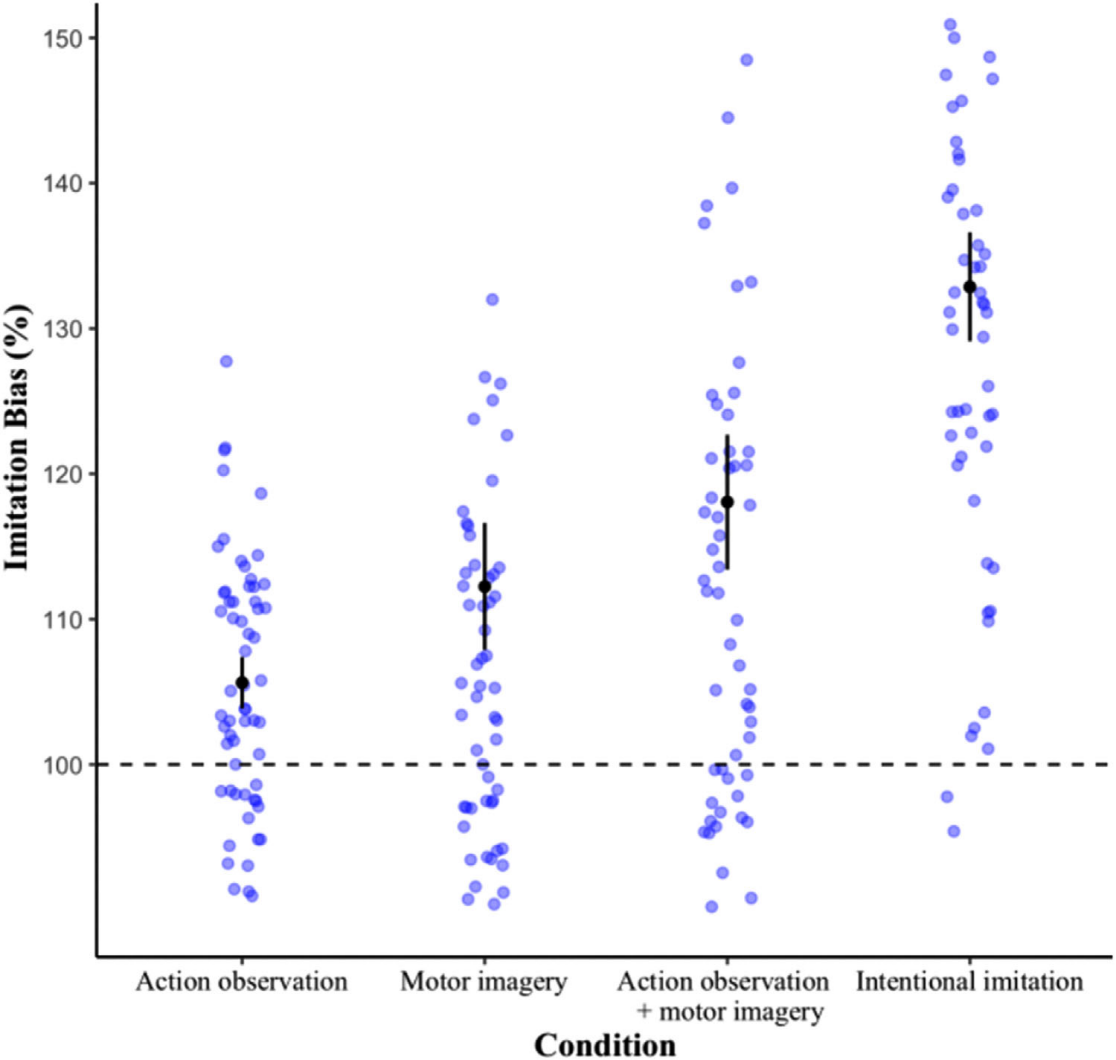
Imitation bias effect (%) across the four instruction conditions with means (black dots) and 95% confidence intervals (black lines). Blue background dots represent mean scores for each participant per block of trials. Although the slow:fast ratio between the two distractor speeds was 150%, a value of 100% represents the absence of an imitation bias effect (dotted black line)

The one‐way ANOVA yielded a significant main effect of instruction (*F*(3, 33) = 14.7, *p* = .01, $\eta_{G}^{2}\;$= 0.44) (see Figure 4). Pairwise comparisons revealed the mean response cycle time ratios, which were calculated across fast and slow trials and from here on referred to as the *imitation bias*, were significantly stronger for AO+MI (118%, 95% confidence interval [CI]: 112.25–123.85), compared to both the AO (106%, 95% CI: 103.42–107.83, *p* = .01) and MI instruction conditions (112%, 95% CI: 106.79–117.71, *p* = .049). The imitation bias was also significantly greater for intentional imitation (133%, 95% CI: 128.17–137.56) than all other conditions (*p* = .01).

The two‐factorial ANOVA (wherein the MI instruction condition was not included) revealed no main effect of compatibility (*F*(1, 13) = 2.52, *p* > .05, $\eta_{p}^{2}$ = 0.16). The main effect of instruction was significant, which only replicates part of the main effect for instruction reported in the aforementioned one‐way ANOVA. The two‐way interaction between compatibility and instruction was not significant.

### 3.2 Neurophysiological data

The two‐factorial ANOVA investigating prefrontal oxygenation showed a significant main effect for instruction condition (*F*(2, 26) = 4.2, *p* = .03), but not hemisphere (*F*(1, 13) = 1.86, *p* = .19). Prefrontal activation was significantly greater overall for the AO+MI instruction (1.26 oxy, 95% CI: 1.02–1.49) when compared to both AO (0.91 oxy, 95% CI: 0.60–1.21, *p* = .01) and MI (0.94 oxy, 95% CI: 0.78–1.11, *p* = .02), but not when compared to the intentional imitation condition (1.05 oxy, 95% CI: 0.87–1.24, *p* > .05). No other comparisons were significant.

**FIGURE 5:**
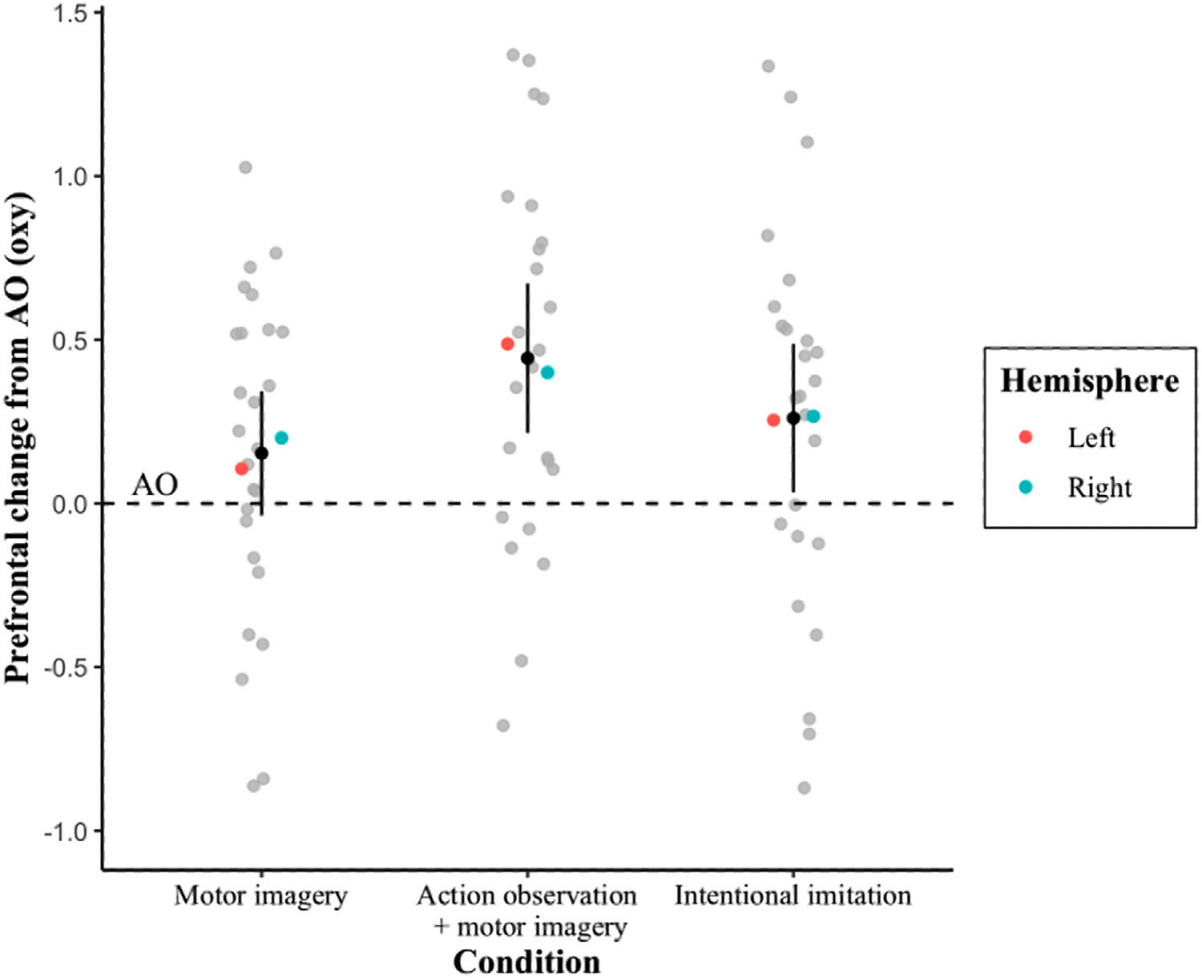
Prefrontal activation (oxy). Mean scores (black dots) with 95% confidence intervals (vertical black lines) are shown for the instruction conditions of MI, AO+MI, and intentional imitation, expressed relative to the AO condition (dotted black line). Overall mean scores are presented for the left and right hemisphere separately (red and blue dots, respectively), whereas individual participant mean scores (gray background dots) are shown for each instruction condition (collapsed across hemisphere)

The two‐way interaction between instruction condition and hemisphere was also significant (*F*(2, 26) = 7.14, *p* < .01) (see Figure 5). In the left hemisphere, pairwise comparisons with Bonferroni corrections applied revealed that the increased oxygenation was significantly higher during AO+MI (1.48 oxy) when compared to both the MI condition (0.85 oxy, *p* = .01) and the intentional imitation condition (1.04 oxy, *p* = .02), but not when compared to the AO condition (0.82 oxy, *p* > .05). No other comparisons were significant. In the right hemisphere, there were no significant differences between any of the instruction conditions: AO, MI, AO+MI, and intentional imitation (0.99, 1.04, 1.03, and 1.07 oxy, respectively). Prefrontal activation was also significantly greater during AO+MI in the left compared to the right hemisphere (*p* = .02).

### 3.3 Perceived closeness data

The self‐report data for the IOS questionnaire were subjected to a one‐way ANOVA examining the main effect of instruction. The MI condition was not assessed via this measure because this instruction did not involve the representation on another person in a movie. The one‐way ANOVA revealed a significant main effect of instruction (*F*(1.7, 18.7) = 10.55, *p* = .01, $\eta_{p}^{2}$ = 0.68). The ratings of perceived closeness between the participant and the actor in the movie were larger in the intentional imitation condition (4.3) than in both the AO+MI (2.9, *p* = .002) and the AO instruction conditions (2.1, *p *= .001). Additionally, the scores for AO+MI were significantly greater than for the AO condition (*p *= .043).

## 4 DISCUSSION

In the present study, we investigated the effects of different motor simulation states on movement kinematics, cerebral oxygenation in the rostral prefrontal cortex, and on self–other perceptions. We predicted that the imitation bias and the perceived closeness would be stronger for combined AO+MI compared to both the AO and MI instructions, and that the related neurophysiological response would be stronger for AO+MI than for AO, MI, and intentional imitation. We also predicted the imitation effect and perceived closeness would be greater for intentional imitation than for the three automatic imitation conditions.

As a crucial prerequisite for interpreting the neurophysiological correlates of AO+MI instructions, the main effect of distractor speed found in the kinematic data replicates our previous results (Eaves et al., 2014; Eaves, Behmer, et al., 2016; Scott et al., 2019), in that the distractor speed effect (i.e., the imitation bias) was significant within all four instruction conditions. Furthermore, the imitation bias was significantly greater for AO+MI than for both AO and MI alone. Although previous research has shown similar results when comparing these conditions across groups of participants over three different experiments (Eaves et al., 2014; Eaves, Behmer, et al., 2016), here we present the first demonstration of this effect within a single experiment using a within‐participants design.

Research that has investigated the neurophysiological effects of AO+MI instructions has previously focused on the neural mechanisms involved in motor planning and motor output (see Eaves, Riach, et al., 2016; Emerson et al., 2018; Vogt et al., 2013). The current study was therefore unique in that we quantified cerebral oxygenation for different motor simulation states (i.e., AO, MI, and combined AO+MI) over the rostral prefrontal cortex in both the left and right hemispheres using fNIRS. These analyses revealed a unique pattern of cerebral oxygenation that was significantly greater for AO+MI than for the other three instruction conditions. In line with both the gateway hypothesis (Burgess et al., 2007, 2006) and our a priori prediction, we interpret this result as tentative evidence in support of participants switching their attentional focus more frequently in the AO+MI condition between external and internal sources of information. In essence, the combined AO+MI instruction requires participants to synchronize the internally generated sensorimotor representation of their imagined action with an externally triggered representation of the observed movement in real time (Emerson et al., 2018). If this is indeed the case, then some form of higher order cognitive control would presumably be needed to maintain the alignment between these two parallel and dynamic motor representations. The present study provides further evidence for the role of the prefrontal cortex within this model of hierarchical cognitive control for dual‐action simulation (Eaves, Behmer, et al., 2016; Eaves, Riach, et al., 2016).

Although a full discussion of prefrontal cortex organization and its roles in executive function is beyond the scope here, the involvement of this region in motor simulation must be considered. Neuroimaging research has previously demonstrated the involvement of the prefrontal cortex across multiple cognitive processes, including attentional shifting, updating and inhibition, working memory, and planning (Collette et al., 2006; Nee et al., 2007; Wager et al., 2004). The motor cognitive model argues executive functions have a central role in motor simulation that is not apparent during overt motor execution (Glover & Baran, 2017). This proposal is derived from evidence (Glover, 2004) demonstrating the differentiation between planning and control of motor actions, which are not accounted for within the prevalent functional‐equivalence model proposed by Jeannerod (2001, 2006). Similar to movement execution, motor simulation requires both preplanning and real‐time execution of imagined actions. The initial motor simulation is generated and constructed from the same internal motor representations that are involved in planning overt actions (Macuga & Frey, 2012). As such, in this phase, motor simulation and action execution neurologically overlap and are considered functionally equivalent (see Decety, 1996; Jeannerod, 1994). Overt action execution, however, involves both unconscious and automatic feedback processes (visual and proprioceptive) as well as predictive forward models for the monitoring and correction of action execution (Cameron et al., 2009; Glover, 2004; Pisella et al., 2000). During pure motor simulation, which is without access to these processes, real‐time monitoring of the simulation relies on a central pool of executive resources, similar to those involved in working memory (Nieuwenstein & Wyble, 2014). Functional equivalence considers that MI should be accurate across a wide range of tasks and conditions. Furthermore, the functional equivalence perspective predicts little difference in neural activity between motor simulation and action execution or systematic timing errors (Glover & Baran, 2017). Because we have found clear differences between the simulation conditions studied in the present study, our data may provide further support aligned with the growing behavioral, neurophysiological, and conceptual evidence for the motor cognitive model of motor simulation, rather than functional equivalence (Glover et al., 2020).

In the present study, we also show for the first time that a combined AO+MI instruction produces significantly greater cerebral oxygenation in the left prefrontal cortex than intentional imitation. This indicates that the cognitive involvement differs between these two conditions. Presumably, intentional imitation does not involve the same degree of attentional switching as AO+MI, perhaps due to an increase in attentional weighting toward external sources of information for motor planning in the intentional imitation condition. Further research is now warranted to explore the differences in the neural involvement between these two conditions in more detail.

fMRI studies have previously shown increased involvement in the left dorsolateral prefrontal cortex during observational learning (e.g., Higuchi et al., 2012). We would argue that this additional activity was not reflected in our fNIRS data for the intentional imitation condition because the types of action we used in the current study were familiar to participants, relatively simple, and therefore most likely already in their motor repertoire. In line with a neural efficiency account of brain processes, we therefore did not predict an increase in the neurophysiological activity in frontal regions during intentional imitation. In addition, we would anticipate activations in other sensorimotor brain areas during intentional imitation conditions that were not assessed in this study (see Rizzolatti & Sinigaglia, 2010). As our fNIRS system was restricted to recording activity over the prefrontal cortex, we cannot confirm this in the present study.

Our results replicate and extend the findings reported in Eaves, Behmer, et al.’s (2016) EEG study. Those authors found that ERD localized over the left rostral prefrontal cortex was significantly greater for AO+MI instructions than in both the AO and MI conditions. In their study, the AO condition did not involve motor execution. The current findings therefore extend Eaves, Behmer, et al.’s (2016) results by showing the same effect occurs even when execution is required after AO, and that this is also detectable using fNIRS as an alternative and low‐cost neuroimaging technique to EEG.

The present findings are also in agreement with a recent TMS study by Bruton et al. (2020). In their study, participants observed index finger abduction–adduction movements while imagining the same action (congruent AO+MI) or little finger abduction–adduction (coordinative AO+MI). When eye gaze behavior was controlled for in the analysis, corticospinal excitability (recorded via motor evoked potentials in the hand) was facilitated in both the congruent and coordinative AO+MI conditions (relative to control conditions) in the muscles that were involved in both the observed and imagined actions. The result for coordinative AO+MI is particularly important, demonstrating that observed and imagined actions can be represented in parallel at a motoric level, even when the content of these two action simulations differs; in this case, different effectors (see Meers et al., 2020 for an alternative account). Although Bruton et al.’s (2020) study provides evidence supporting the dual‐action simulation hypothesis, in the current study we extended this approach further to investigate the neural substrates of cognitive control during dual‐action simulation. Future research should now explore how different AO+MI states, which can range from congruent, across coordinative, to conflicting AO+MI, may impact indicators of cognitive load and the associated activity in underlying brain regions. Indeed, Bruton et al. (2020) included a social validation report where participants identified varying cognitive demands for the different AO+MI states via introspection.

An additional and unexpected finding was that cerebral oxygenation was significantly right lateralized during the MI instruction. This was surprising because the activity across motor and premotor areas is typically increased in the contralateral hemisphere during MI relative to control conditions (Hétu et al., 2013). However, the right dorsolateral prefrontal cortex is an area sensitive to individual motor impulsiveness (Asahi et al., 2004). This activity may therefore reflect the capacity for overt response inhibition during internal motor simulation.

The capacity to differentiate between the self and other people is essential for inhibiting the automatic tendency to imitate observed actions (Brass et al., 2009). This is because the observer must continuously distinguish between their own motor plans and the actions of observed agents (see Brass & Heyes, 2005). In the present study, the IOS scale was used as a nonspecific measure for assessing the general sense of being interconnected (i.e., closeness) with another person. This can be conceptualized in terms of the overlapping representations of the self and the interacting partner. The ratings of perceived closeness between the self and other were significantly larger for AO+MI than for AO, with intentional imitation producing significantly greater values overall. These findings reveal a significant modulatory effect of the instruction condition (and therefore the related motor simulation state) on self–other perceptions. This in part further aligns with the current understanding of the rostromedial prefrontal cortex as a widely distributed neural network, additionally including both right and left temporoparietal junction as well as the precuneus (BA 7). This network is involved in many perceptual, motor, affective, and cognitive functions, such as mental imagery, theory of mind, self‐awareness, integrating perceptions, spatial mapping, guiding motor responses, and metacognition (Marek & Dosenbach, 2018). Furthermore, Taube et al. (2015) evidenced significantly greater activity during AO+MI when compared to AO, MI, and also compared to the total sum of AO and MI activity combined within the bilateral precuneus (BA 7). Along with the BA 10, this area is known for its involvement in imagery, perspective taking, and agency (Cavanna & Trimble, 2006), as well as its role in the frontoparietal circuit of attention awareness (Goldstein‐Piekarski & Williams, 2019), and as a correlate of cognitive monitoring (Manna, et al., 2010).

The effect of the instruction condition on self–other perceptions may support a novel theory of social cognition described by Santiesteban et al. (2012). This suggests that self–other discrimination mechanisms also support imitation inhibition. Moreover, the frontal lobes have previously been associated with inhibition of responses to external stimuli (Aron et al., 2003; Konishi et al., 1998; Rubia et al., 2001). Increased activation of medial BA 10 has also been demonstrated using fNIRS in studies that manipulate higher versus lower self‐monitoring components (Herrmann et al., 2003; Kreplin & Fairclough, 2013). Those studies have shown that an increase in self‐monitoring can produce an increase in cerebral blood oxygenation in the medial rostral prefrontal cortex. Within the current study, greater requirement of self‐monitoring may be involved when synchronizing and maintaining the alignment between two parallel and dynamic motor representations (i.e., AO+MI), resulting in more pronounced automatic imitation effects. This is further supported by studies showing participants who score high in “self‐monitoring” (Snyder, 1974), or who have a symbiotic self‐construal, are more likely to mimic others, which suggests the existence of unconscious affiliation strategies (Ainley et al., 2014; Cheng & Chartrand, 2003; Obhi et al., 2011). Furthermore, priming participants with interdependent self‐construal results in increased amplitude of MEPs provoked by TMS (Obhi et al., 2011), which further suggests that these top‐down influences increase cortical excitability in the motor areas that impact imitation.

The task in the current study required participants to execute a prespecified (instructed) action response following the observation of either the same or a different action. Recently, the involvement of domain‐general cognitive control processes has been considered in both preparatory (Cross & Iacoboni, 2014) and reactive modulation of mirroring (Cross et al., 2013). These suggestions align with automatic imitation inhibition in relation to either input or output modulation (Heyes, 2011). Input modulation refers to mediating the processing of action stimuli, whereas output modulation relates to the motor activations associated with this input, which are either inhibited or permitted to influence overt motor responses. Essentially, attentional effects modulate input and social cognitive factors relate to output modulation (see Heyes, 2011).

Although research has investigated self–other distinctions in relation to automatic imitation (Brass & Heyes, 2005), research has not yet comprehensively examined the differences that may arise in population groups with impaired or distorted self–other processing and agency. For example, within patients diagnosed with schizophrenia there is a lack of self‐awareness over the intention of actions. Frith et al. (2000a) argues that in this particular population, the predicted sensory feedback is also altered, which is associated with a failure to form accurate representations of predicted sensory outcomes. Accordingly, this can result in an impaired capacity to differentiate between actions performed by the self or others (Frith et al., 2000b). A study by Enticott et al. (2008), using the TMS methods of Fadiga et al. (1995), specifically examined mirror neuron activity in schizophrenia. This study found a reduced mirror response within this population (i.e., less cortical excitability underlying observation of actions), compared to the “healthy” control group during the AO condition. Their finding provided further evidence of an overall reduced level of mirror neuron activity in schizophrenia. In the context of the current study, future research should examine the potential impact of combined AO+MI instructions in these populations on self–other distinctions and different forms of imitation.

The combined use of AO+MI instructions during intentional imitation should now be explored. This is to determine if such an approach can offer behavioral and/or neurophysiological advantages, beyond those offered via either AO+MI or intentional imitation alone. When considering the AO+MI instruction within practical coaching applications, research should further explore the benefits of integrating the combined AO+MI instruction with traditional intentional imitation instructions (McNeill et al., 2019). This would provide a greater ecological test of combined AO+MI instructions. Although research shows AO+MI training can have positive effects on motor skills in neurodevelopmental populations, such as children with developmental coordination disorder (Marshall, Wright, Holmes, Williams, et al., 2020; Scott et al., 2019, 2020) and in stroke rehabilitation (Sun et al., 2016), the potential for changes in perceived interconnectedness in psychiatric conditions remains an unexplored question. In line with previous studies using the same paradigm, the main effect of compatibility was not significant in both the AO+MI condition (e.g., Eaves et al., 2014; Eaves, Riach, et al., 2016) and the intentional imitation condition (e.g., Eaves et al., 2012).

## 5 CONCLUSIONS

The current study replicates previous results (Eaves et al., 2014; Eaves, Behmer, et al., 2016; Scott et al., 2019) demonstrating a significantly stronger imitation bias for the AO+MI instruction than for AO and MI individually. Although previous research has demonstrated the same effect using different groups of participants across different studies (Eaves et al., 2014, 2012), here we reproduce this effect using a within‐participants design. These behavioral findings were a crucial prerequisite for interpreting the neurophysiological correlates of the AO+MI instruction. The current study uniquely quantified neurophysiological markers of different motor simulation states over the left rostral prefrontal cortex using fNIRS. This revealed cerebral oxygenation in this brain region is significantly greater during combined AO+MI than during all other instructions, including intentional imitation. In line with the gateway hypothesis (Burgess et al., 2007, 2006), we interpret this as evidence of switching attentional focus more frequently in the AO+MI condition between external and internal sources of information. The pattern of left prefrontal activation recorded in the present study may therefore identify a neural signature of AO+MI, supporting attentional switching between concurrent representations of self (MI) and other (AO), to increase imitation and perceived closeness. We contend that this activity indicates a role for this brain region in higher order cognitive control, which is presumably necessary to maintain the alignment between two parallel and dynamic motor representations. The present study paves the way for further research into the role of the prefrontal cortex within this model of hierarchical cognitive control for dual‐action simulation.

### PEER REVIEW

The peer review history for this article is available at https://publons.com/publon/10.1002/brb3.2407.

## Data Availability

The data that support the findings of this study will be made openly available in the reserved repository Emerson, JR (2021), “Prefrontal involvement in motor simulation states,” Mendeley Data, v1. Available at: https://doi.org/10.17632/9jw2b93kns.1.
